# Complete chloroplast genome sequences of *Corydalis edulis* and *Corydalis shensiana* (Papaveraceae)

**DOI:** 10.1080/23802359.2020.1863167

**Published:** 2021-01-27

**Authors:** Yan-Yan Liu, Sheng-Long Kan, Jun-Li Wang, Ya-Nan Cao, Jia-Mei Li

**Affiliations:** a College of Plant Protection, Henan Agriculture University, Zhengzhou, China; b College of Life Sciences, Henan Agriculture University, Zhengzhou, China; c State Key Laboratory of Systematic and Evolutionary Botany, Institute of Botany, Chinese Academy of Sciences, Beijing, China

**Keywords:** Chloroplast genome, *Corydalis*, Papaveraceae

## Abstract

*Corydalis* DC., the largest genus of Papaveraceae, was recognized as one of the most taxonomically challenging plant taxa. Due to the lack of genetic information used in previous studies, species discrimination and taxonomic assignment in *Corydalis* have not been fully solved. Here, the complete chloroplast genomes were reported for *Corydalis edulis* Maxim. and *Corydalis shensiana* Liden, with their genome sizes being 154,395 and 155,938 bp, respectively. Both of the chloroplast genomes comprise two inverted repeat (IR) regions, separated by a large single-copy (LSC) region and a small single-copy (SSC) region, and encode 130 genes, including 85 protein-coding genes, 8 ribosomal RNA genes, 37 transfer RNA genes. Our study will provide novel insight into the molecular phylogeny and classification of *Corydalis*.

*Corydalis* DC., the largest genus of Papaveraceae, contains about 400 species (Zhang et al. 2008). This genus is an important component of the biodiversity in the Himalaya–Hengduan Mountains and was recognized as one of the most taxonomically challenging plant taxa. Due to the lack of genetic information used in previous studies, species discrimination and taxonomic assignment in *Corydalis* have not been fully solved (Wang 2006; Ren et al. 2019). In recent years, the whole chloroplast (cp) genomes have become valuable resources for molecular phylogeny and species identification due to the maternal mode of inheritance, dense gene content, and slower evolutionary rates relative to those of nuclear and mitochondrial genomes (Wicke et al. 2011). In this study, we reported the complete cp genomes of *Corydalis edulis* Maxim. and *C. shensiana* Liden, which will provide novel insight into the molecular phylogeny and classification of *Corydalis*.

The fresh leaves of *C. edulis* and *C. shensiana* were collected from Nanyang, Henan Province, China (E111°15′41′′, 33°25′1′′) and Fengxian, Shaanxi Province, China (E106°36′27′′, N34°12′21′′), respectively. The voucher specimens were deposited in Henan Agricultural University Herbarium (LYY1933001 and LYY19051101). Total genomic DNA was extracted from silica gel-dried leaves with the CTAB method (Rogers and Bendich 1988) and sequenced using Illumina Hiseq2500 platform at Suzhou Jinweizhi Biotechnology Institute. In total, 42.1 and 39.6 million (M) high-quality raw reads (150 bp PE read length, with Q30 > 91%) were generated for *C. edulis* and *C. shensiana*, respectively. The raw reads were filtered using CLC Genomics Workbench (http://www.clcbio.com) to remove low-quality reads and those containing adaptors with the default settings. The clean reads were assembled into the draft cp genome by CLC Genomics Workbench and GENEIOUS V11.01 (http://www.geneious.com) with *Coreanomecon hylomeconoides* Nakai as the reference genome (GenBank accession number: NC_031446.1). The assembled cp genomes were annotated using PGA (Plastid Genome Annotator) (Qu et al. 2019). To validate the assembly, PCR amplifications and sanger sequencing were performed to confirm the four junction regions between inverted repeat (IRs) and large single-copy region (LSC)/ small single copy region (SSC) and the region with great difference with the reference. Then, the start/stop codons and intron/exon boundaries of genes were subsequently manually modified based on the reference sequences, and the online program OGDRAW (OrganellarGenomeDRAW) (Greiner et al. 2019) was used to generate the graphical genome map of the cp genomes.

The full length of *C. edulis* cp genome (GenBank accession number: MW110633) was 154,395 bp and comprised of an LSC (82,391 bp), an SSC (19,504 bp), and two IRs (26,250 bp, each). And the complete cp genome of *C. shensiana* (GenBank accession number: MW110634) was 155,935 bp in length and contained two IRs (26,344 bp, each), an LSC (82,752 bp), and an SSC (20,495 bp). The overall GC content of *C. edulis* and *C. shensiana* cp genomes were 40.24% and 40.57%, respectively. Both of the two cp genomes contained 130 genes, including 85 protein-coding genes (*ycf1* and *ycf2* are two pseudogenes, and *rps16* or *clpP* are partial sequence), 8 ribosomal RNA genes, 37 transfer RNA genes. Of those protein-coding genes, 9 (*atpF*, *ndhA*, *ndhB*, *petB*, *petD*, *rpl2*, *rpl16*, *rpoC1*, and *rps16*) contained one intron and 3 (*clpP*, *rps12*, and *ycf3*) contained two introns. The overall structure, gene content, and arrangement of the cp genomes of *C. edulis* and *C. shensiana* were quite similar to but with higher quality than two previously reported *Corydalis* species, in which several subunits of NADH-dehydrogenase genes were absent or with partial sequence (such as *ndhC*, *ndhD*, *ndhF*, and *ndhI*) (Kanwal et al. 2019).

Sixteen cp genomes of Papaveraceae were fully aligned with MAFFT v7.3 (Katoh and Standley 2013), and the maximum-likelihood (ML) tree was constructed using all coding sequences under the GTRGAMMA model with 100 bootstrap replicates by RAxML v.8.2.1 (Stamatakis 2014). *Euptelea pleiosperma* J. D. Hooker & Thomson was chosen as an outgroup. The result showed that the four *Corydalis* species (*C. edulis*, *C. shensiana*, *C. trisecta* and *C. conspersa*) are strongly supported as monophyletic (Figure 1).

**Figure 1.**
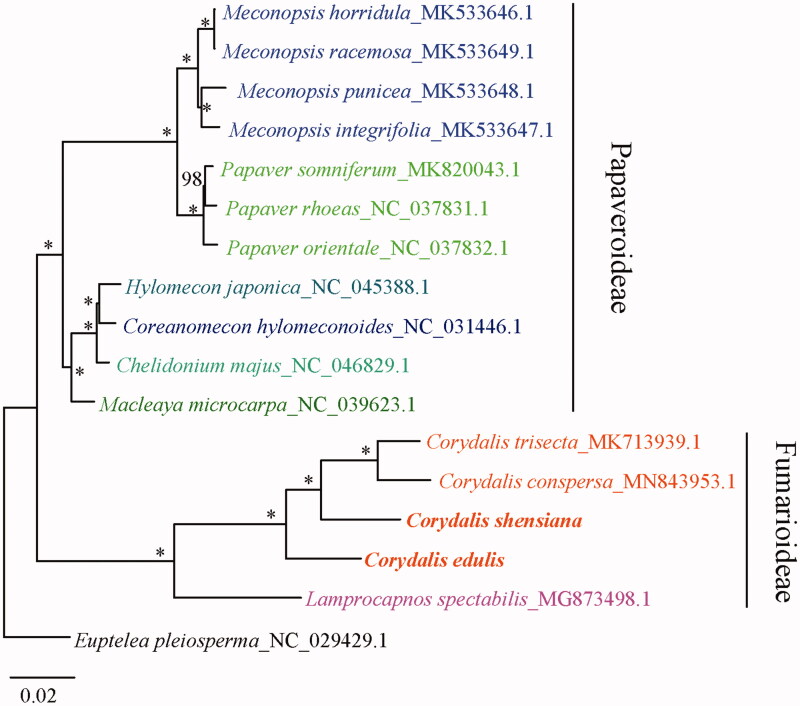
ML tree inferred from the coding sequences of 16 chloroplast genomes in Papaveraceae. Numbers above each node indicate bootstrap values. The asterisks show nodes supported by a bootstrap value of 100%.

## Data Availability

The assembled cp genomes of this study are openly available in NCBI at https://www.ncbi.nlm.nih.gov/WebSub/?form=history&tool=genbank, reference number (MW110633 and MW110634). The raw data that support the findings of this study are available on request from the first author LYY. The data are not publicly available due to their containing information that could compromise the privacy of research participants.
